# Chemical, Enantioselective, and Sensory Analysis of a Cholinesterase Inhibitor Essential Oil from *Coreopsis triloba* S.F. Blake (Asteraceae)

**DOI:** 10.3390/plants8110448

**Published:** 2019-10-25

**Authors:** Sandra Espinosa, Nicole Bec, Christian Larroque, Jorge Ramírez, Barbara Sgorbini, Carlo Bicchi, Gianluca Gilardoni

**Affiliations:** 1 Departamento de Química y Ciencias Exactas, Universidad Técnica Particular de Loja, 1101608 Loja, Ecuador; sandraespinosa100@hotmail.com (S.E.); jyramirez@utpl.edu.ec (J.R.); 2 Institute for Regenerative Medicine and Biotherapy (IRMB), Inserm U1183, CHRU of Montpellier, 34295 Montpellier, France; nicole.bec@inserm.fr; 3 Supportive Care Unit, Institut du Cancer de Montpellier (ICM), 34298 Montpellier, France; cjlarroque@gmail.com; 4 Dipartimento di Scienza e Tecnologia del Farmaco, Università degli Studi di Torino, 10125 Torino, Italy; barbara.sgorbini@unito.it (B.S.); carlo.bicchi@unito.it (C.B.)

**Keywords:** *Coreopsis triloba*, *Coreopsis capillacea*, essential oil, GC-FID/GC-MS, GC-O, AEDA, AChE, BChE, Ecuador

## Abstract

The fresh leaves of *Coreopsis triloba* S.F. Blake, collected at Cerro Villonaco in Loja, Ecuador, were investigated with respect to their essential oil (EO). The chemical composition was determined qualitatively through gas chromatography coupled with mass spectrometry (GC-MS) and quantitatively by gas chromatography coupled with flame ionization (GC-FID), using relative response factors (RRF) based on the enthalpy of combustion. The essential oil contained between 92.5% and 93.4% of monoterpene hydrocarbons, with (*E*)-*β*-ocimene being the main component (35.2–35.9%), followed by *β*-phellandrene (24.6–25.0%), α-pinene (15.3–15.9%), myrcene (10.9–11.0%), sabinene (2.2–2.4%), (*Z*)-*β*-ocimene (1.5%), and germacrene D (1.2–1.3%). The enantiomeric distribution of α-pinene, *β*-pinene, limonene, and germacrene D was also determined. The main components responsible for the aroma were identified through aroma extract dilution analysis (AEDA), a gas chromatography-olfactometry (GC-O) based technique, being *α*-pinene, *β*-pinene (0.6%), terpinolene (0.1%), *α-*copaene (0.1–0.3%), *β-*phellandrene, and (*E*)-4,8-dimethyl-1,3,7-nonatriene (0.1–0.2%) the main olfactory constituents according to the decreasing factor of dilution (FD) order. The biological tests showed IC_50_ inhibition values of 42.2 and 6.8 µg/mL for acetylcholinesterase (AChE) and butyrylcholinesterase (BChE), respectively.

## 1. Introduction

Ecuador is a country of impressive botanical abundance, where the traditional use of many plant species has attracted the curiosity of researchers of natural products. The study of medicinal plants revealed the existence of secondary metabolites, responsible for their biological activity [1,2,3,4]. Among the pharmacologically interesting natural products obtained by plants we can cite essential oils. They are products that are representative of a part of the volatile fraction, obtained by steam or hydrodistillation [5], and consisting mainly of mono- and sesquiterpenoids [6], often characterized by interesting biological properties [7]. Nowadays, essential oils are important complements to allopathic treatments of various diseases [8,9,10], justifying the interest in studying these products obtained from plants used in traditional medicine. Actually, most EOs are just described for their antibacterial and antimycotic properties, which are often applicable only as antiseptic formulations or food preservatives. However, it is the opinion of the authors that the description of less common biological activities could greatly expand their application and contribute to improving the knowledge on the pharmacology of secondary metabolites. For this reason, the cholinesterase enzymes could be interesting uncommon targets for the EOs to extend the investigation of these products to the treatment of Alzheimer’s disease. Some evidence in this sense is already present in the literature [11], where a good inhibition activity has been reported for EOs of prevalent monoterpenic composition, confirming that AChE and BChE inhibition by EOs is a possible and, until now, under-explored field of investigation.

Alzheimer’s disease (AD) is a chronic neurodegenerative illness characterized by a progressive deterioration of memory and other cognitive functions and is currently the focus of scientific research aimed at finding effective therapeutic strategies, mainly related to the inhibition of the enzymes acetylcholinesterase and butyrylcholinesterase, known as cholinesterases (ChEs). These enzymes are involved in the development of the disease, since they catalyze the degradation of the neurotransmitter acetylcholine (ACh) [12]. As summarized in the 2016 World Alzheimer’s Report, 47 million people live with dementia worldwide, and it is estimated that this number will increase to more than 131 million by the year 2050. Research focused on the administration of ChE inhibitors has shown that they increase the levels of ACh in the brain [13,14], counteracting the progress of the disease. The current discussion concerning the effectiveness and costs of drugs in the clinic for the treatment of AD [15,16] is the basis for investigations of natural products [14] and for the traditional treatments of so called “head” diseases.

*Coreopsis triloba* S.F. Blake, also cited as *Coreopsis capillacea* (Kunth), is a species belonging to the Asteraceae family, the richest angiosperm family. It is a shrub that is 0.5–1.2 m tall and often densely branched. This species is distributed in the countries of Ecuador (1400–2800 m) and Peru (1400–2500 m), being particularly common in the province of Loja (Ecuador). The species is also known with the following Quechua names: *Macchia*, *peña nachic*, and *caca nachic*, and it is traditionally used in medicinal baths for treating inflammation [17].

The only study of an essential oil from this genus that reports on the chemical composition and antioxidant activity of the fraction distilled from *Coreopsis tinctoria* Nutt., with limonene (11.31%), *α*-bergamotene (7.31%), and *α*-phellandrene (3.88%) being the main components [18]. Many other studies are present in the literature on *C. tinctoria*, however, all of them refer to the non-volatile fraction. *C. lanceolata*, *C. grandiflora*, *C. mutica* and many other species of this genus have also been described in the literature for the chemical composition of their non-volatile fractions [19,20].

To the best of the author’s knowledge, this is the first article that reports on the chemical composition and the cholinesterase inhibition activity of *C. triloba* essential oil and on the chemistry of this species in general.

## 2. Results

### 2.1. Chemical Analysis

The essential oil yield of *C. triloba* S.F. Blake is 0.1% ± 0.03% (w/w) for both the hydrodistillation and steam distillation on fresh plant material.

Table 1 lists the identified components together with their relative percent abundance, calculated vs. nonane as the internal standard. The major components were (*E*)-*β*-ocimene (35.2–35.9%), followed by *β*-phellandrene (24.6–25.0%), α-pinene (15.3–15.9%), myrcene (10.9–11.0%), sabinene (2.2–2.4%), (*Z*)-*β*-ocimene (1.5%), and germacrene D (1.2–1.3%). In addition to the listed components, traces or low amounts (0.1–0.6%) of ten unidentified sesquiterpenes have been detected.

### 2.2. Enantioselective Analysis

The enantiomeric composition of chiral components (Table 2) were obtained by enantioselective GC, carried out with diethyl tertbutylsilyl-β-cyclodextrin as the chiral stationary phase [29], and identified by comparing the linear retention index of each separated enantiomer with enantiomerically pure standards. (+)-β-Pinene and (−)-germacrene D were present as pure enantiomers, while (S)-α-pinene and (S)-limonene presented enantiomeric excesses.

### 2.3. AEDA GC-O

**Figure 1:**
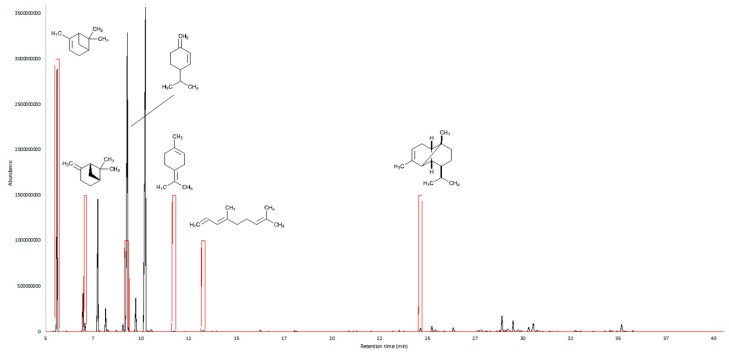
Superposition of the gas chromatogram and the aromagram of the essential oil.

**Figure 2:**
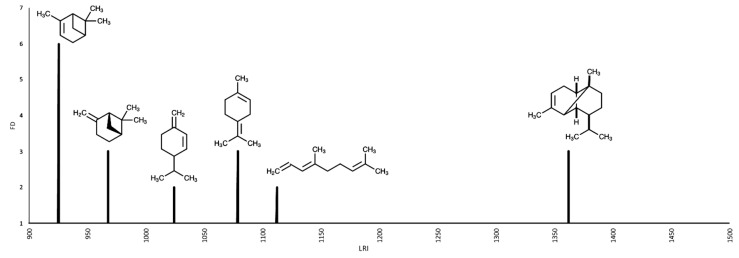
Aromagram of the essential oil of *Coreopsis triloba* S.F. Blake.

GC-O results were obtained with the AEDA method and are shown in Figure 1 and Figure 2, and in Table 3. Six main compounds responsible for the *C. triloba* essential oil aroma were detected. The most important sensory component, determined with the AEDA GC-O analysis, was α-pinene, showing a strong woody odor, followed by β-pinene, terpinolene, and α-copaene. 

### 2.4. Cholinesterase Inhibition Test

**Figure 3:**
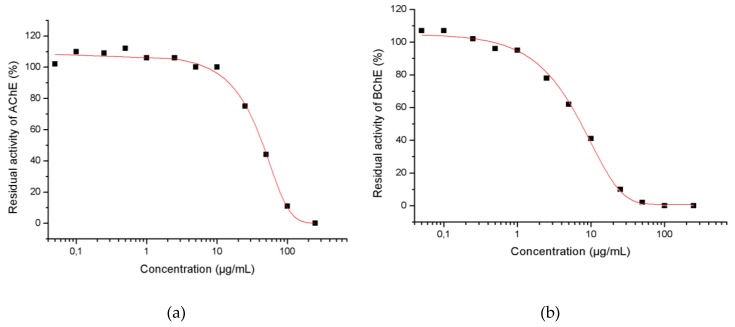
IC_50_ values for *Coreopsis triloba* S.F. Blake essential oil vs. (**a**) acetylcholinesterase (AChE) and (**b**) butyrylcholinesterase (BChE). IC_50_: half maximal inhibitory concentration.

The inhibitory activity of *Coreopsis triloba* S.F. Blake essential oil against AChE and BChE shows IC_50_ concentrations of 42.2 and 6.8 μg/mL, respectively (Table 4 and Figure 3). These results were compared with Donepezil inhibition values, which show an IC_50_ value of 0.04 μg/mL on AChE and 3.6 μg/mL on BChE. The comparison of the inhibition of the *C. triloba* essential oil versus the positive control indicated that its activity is three thousand times lower on AChE and two times lower on BChE than Donepezil.

The inhibitory activity of *Coreopsis triloba* S.F. Blake essential oil has not been previously described. The high inhibition found makes this a promising essential oil for further studies on the relationship between composition and bioactivity and for the possible development of more bioactive derivatives against neurodegenerative diseases.

## 3. Discussion

In this study, two different forms of distillation have been applied: a classical steam distillation process and micro-scale hydrodistillation. The first method was used in order to obtain a discrete amount of pure essential oil, necessary to perform GC-O analyses and biological essays. In fact, in the AEDA experiments, relatively high concentrations were needed due to the lower sensitivity of the human nose to odor perception and to the necessity of performing many repeated injections. As concerns the biological tests, we needed to dilute exactly the essential oil in DMSO and perform different dilutions to achieve the final results. On the other hand, this plant is not very common and it was not possible to collect the vegetal material in such an amount as to perform the preparative repetitions that are necessary to obtain a significant quantitative result. Hence, we decided to use a micro-scale Marcusson-type apparatus, with which hydrodistillation can be performed with a few grams of plant material, obtaining 400 μL of a hexane solution of EO with the internal standard, suitable for quantitative analysis. Theoretically, steam distillation and hydrodistillation, although they are based on the same physical principle, can afford a different quantitative result, due to the different partial water-solubility of the EO constituents. In particular, the Marcusson apparatus includes a solvent extraction step, where water-soluble compounds can be extracted increasing their amount in the mixture. However, in this study the comparison of the EOs distilled in both ways resulted in being quite similar. We could explain this phenomenon, as the hydrocarbon prevalent composition resulted in a very apolar mixture, where water solubility of the constituents was actually negligible or, at least, quite constant for all the main ingredients. The overall chromatographic profile resulted in being qualitatively and quantitatively quite similar, justifying the use of both volatile fractions in the study.

Currently, the enantiomeric ratio of an essential oil is considered the important information obtained from its chemical analysis [30,31,32]. The importance has been marked by the relationship of the enantiomeric composition with organoleptic properties, the clarification of the biosynthetic pathway of a metabolite, the origin, the quality, and the possible adulteration, as well as its biological activity [33,34,35]. The monoterpenes α-pinene, β-pinene, and terpinolene have been characterized as important components responsible for the odor in a large number of plant species [36,37,38,39,40]. The sesquiterpene α-copaene has also been reported in olfactory profile investigations, although not frequently [41]. Some compounds resulted with similar odor perceptions, such as *β*-phellandrene and terpinolene, in which a mild herbal odor was perceived, while α-pinene, *β*-pinene, and α-copaene had a woody or pine smell and only (*E*)-4,8-dimethyl-1,3,7-nonatriene showed a spicy odor.

Although ACh can be hydrolyzed by both AChE and BChE, it has been found that AChE plays the major role in the hydrolysis of ACh in the healthy brain, whereas BChE takes over hydrolysis of ACh in the deficient AChE brain, it has been shown according to tests in mice [9]. However, the inhibition of BChE is considered a potential therapeutic target to restore the levels of ACh in the brain, improving cognitive deterioration and reducing the adverse effects in patients with Alzheimer’s disease. The absence of AChE does not result in an increase of ACh concentration, because BChE takes over its degradation [42]. Furthermore, the higher activity of the EO toward BChE can be interpreted as a selective enzymatic inhibition, permitting to imagine the existence of a specific inhibitory mechanism for some constituent of the mixture. This phenomenon, far from being negative, can be of pharmacological interest and does not reduce the importance of the result.

## 4. Materials and Methods

### 4.1. Materials and Equipment

Qualitative analyses were run with a GC-MS system consisting of an Agilent Technologies gas chromatograph 6890N and a quadrupole mass spectrometry detector 5973 (Santa Clara, CA, USA), operating in SCAN mode and electronic ionization (70 eV), with a mass range of 45–350 m/z. The gas chromatograph was equipped with a non-polar stationary phase capillary column DB-5MS (Agilent Technologies) (5%-phenyl-methylpolysiloxane, 30 m, 0.25 mm internal diameter and 0.25 μm film thickness; J; W Scientific, Folsom, CA, USA) and a HP-INNOWAX polar stationary phase column (Agilent Technologies) (polyethylene glycol, 30 m, 0.25 mm internal diameter and 0.25 μm film thickness; J; W Scientific, Folsom, CA, USA).

Quantitative analyses were carried out with a GC-FID system consisting of an Agilent Technologies gas chromatograph 6890N (Santa Clara, CA, USA) with an Agilent Technologies 7683 series autoinjector (Little Falls, DE, USA). Linear retention indices were obtained using the homologous series of linear alkanes (C_9_ from BDH, purity 99% and C_10_–C_25_ from Fluka, purity 99%).

The enantiomeric components and excesses (e.e.) were determined using an enantioselective column diethyl tertbutylsilyl-β-cyclodextrin (25 m × 0.25 mm × film thickness 0.25 µm) from Mega (Legnano, MI, Italy).

The GC-O analyses were carried out on an Agilent Technologies gas chromatograph 6890N (Santa Clara, CA, USA) coupled with a FID and a GERSTEL ODP 3 olfactory detection port 07615-00113. The analyses were performed with the same DB-5MS column and automatic injector previously described. The GC-O system was configured with a split fraction between sniffing port and detector of 50%.

All solvents used in this study were of analytical grade (purity > 99%) from Sigma–Aldrich. 5,5′-Dithiobis (2-nitrobenzoic acid), DNTB (Sigma–Aldrich), dimethyl sulfoxide, DMSO (Sigma–Aldrich), *Εlectrophorus electricus* acetylcholinesterase (Type VS, freeze-dried powder, 744 U/mg solid, 1272 U/mg protein), equine serum butyrylcholinesterase (lyophilized powder, 900 units/mg protein), acetylthiocholine iodide (Sigma–Aldrich) and a Varioskan Flash (Thermo Scientific) detection system were used for enzymatic inhibition experiments. Donepezil was taken as positive control of ChE inhibition.

### 4.2. Plant Material

The aerial parts of *Coreopsis triloba* S.F. Blake were collected in the province of Loja on Mount Villonaco, at an altitude of 2724 meters above sea level, with geographic coordinates 690644E–9557656N. The plant was collected with permission of the Ministry of Environment of Ecuador (MAE-DBN-2016-0655) and the identification was achieved by Dr. Nixon Cumbicus and Dr. Vladimir Morocho of the Universidad Técnica Particular de Loja (UTPL). A voucher specimen of the species was deposited in the UTPL herbarium with code VMZ_011.

### 4.3. Isolation of the Essential Oil

The pure essential oil was obtained by steam distillation for 4 h [43] from 2 kg of the fresh aerial parts. This product was used for sensory and biological analyses. 

For the analytical purpose, the essential oil was obtained by hydrodistillation. Four repetitions were carried out using a micro Marcusson-type apparatus [44,45]. Five grams of fresh plant material were distilled for 90 min and the essential oil collected in 500 μL of cyclohexane, containing *n*-nonane as internal standard (0.7 mg/mL).

During the analyses, all samples were stored in amber vials at −15 °C. The yield was directly calculated by weight in the case of the pure essential oil and analytically, as average and standard deviation, in the case of the micro-scale distillations.

### 4.4. Qualitative Chemical Analysis

The following operative conditions were applied for GC-MS analysis with DB-5MS column: He was the carrier gas, with a flow rate of 1 mL/min; the injection volume was 1 μL. The injector was operated in split mode, with a split ratio of 40:1. The injection temperature was set at 220 °C. The elution was conducted according to the following temperature program: 50 °C for 5 min, a first thermal gradient to 180 °C at a rate of 3 °C/min, then a second gradient to 250 °C at a rate of 15 °C/min. At the end, the oven temperature was kept at 250 °C for 15 min. 

With the HP-INNOWAX column, the same conditions and thermal program as DB-5MS were applied, with the exception of the final oven temperature that was set at 230 °C.

The linear retention indices were obtained according to Van Den Dool and Kratz [46].

### 4.5. Quantitative Chemical Analysis

The quantitative analyses were carried out with the same instrumental conditions as the qualitative ones. The FID was alimented as follows: hydrogen flow 30 mL/min, air flow 300 mL/min. The temperature of the detector was set at 250 °C. The quantitative composition was obtained by using relative response factors calculated on the basis of the combustion enthalpy [47,48]. The original method was modified, since isopropyl caproate instead of methyl octanoate was used as a calibration standard. Isopropyl caproate was obtained in the authors’ laboratory by synthesis and its GC purity was determined as 97% (GC). Furthermore, a calibration curve was constructed for each column. Six calibration standard dilutions were taken to build-up the calibration curve, corresponding to 0.6, 1.8, 4.3, 8.3, 16.8, and 34.3 mg of isopropyl caproate in 10 mL of cyclohexane respectively. Each solution contained 7.0 mg of nonane as internal standard. The calibration curve corresponding to the analysis with the DB-5MS column generated a correlation coefficient of 0.9997, while in the HP-INNOWAX analysis it was 0.9991.

### 4.6. Enantioselective GC Analysis

Enantioselective GC-MS analysis was performed under the following oven thermal program: 50 °C, held for 5 min, rising to 220 °C at a rate of 2 °C/min and kept at this temperature for 5 min. The elution order was established by injecting enantiomerically pure standards, available in one of the authors’ laboratories (C.B.).

### 4.7. GC-O Analysis

The system was operated according to the following thermal gradient: 50 °C held for 1 min, rising at 2 °C/min to 100 °C, then to 200 °C (1 min) at a rate of 20 °C/min. The carrier gas (He) and detector were set at the same conditions as for the quantitative analysis. 

The samples were prepared as solutions of the essential oil in cyclohexane at the concentration of 200 μL/mL, corresponding to a dilution factor (FD) of 1; the injection volume was 1 μL. The AEDA method was applied by acting on the split ratio (splitless, 1:1, 2:1, 3:1, 4:1, 5:1, and 6:1). The sniffing procedure was divided into 30 min sessions with 20 min intervals to avoid lassitude. The GC-O analyses, executed in duplicate, were performed by two analysts, presenting no anosmia for common terpenes and with previous training and experience in GC-O perception applied to essential oils. The acceptance criteria for the detected odors was that each perception had to be confirmed by at least one panelist in, at least, two following dilutions or, alternatively, once by both panelists at the same dilution.

### 4.8. Cholinesterase Inhibition Test

The activities of cholinesterase (ChE) were evaluated by following a colorimetric protocol adapted from Ellman et al. [49]. The catalyst efficiently hydrolyzes acetylthiocholine (ATCh), which is the sulfur analogue of the natural substrate of these enzymes. After hydrolysis, this substrate analogue produces acetate ion and thiocoline. Thiocoline, in the presence of the highly reactive dithiobisnitrobenzoate ion (DTNB), produces a yellow color, which can be monitored quantitatively by measuring its spectrophotometric absorption at 412 nm. The inhibition assay volume contained 200 μL of phosphate buffered saline (pH 7.4), DNTB (1.5 mM) and test sample diluted in DMSO (1% v/v). Both *Electrophorus electricus* acetylcholinesterase and equine serum butyrylcholinesterase were dissolved in PBS pH 7.4 and were used at 25 mU/mL for the assay. After 10 min of preincubation, the substrate acetylthiocholine iodide (1,5 mM) was added to start the reaction. During 30 min of incubation at 30 °C, multiple 96-well microliter sites were read in a Varioskan Flash (Thermo Scientific) detection system. All measurements were made in triplicate. When possible, the IC_50_ values were calculated using the GNUPLOT package online (www.ic50.tk, www.gnuplot.info). Donepezil was used as reference ChE inhibitor with an IC_50_ = 100 nM for AChE and 8500 nM for BChE. In this assay, the possibility of false positive inhibition results previously described for the high concentration (>100μg/mL) of amine or aldehyde compounds cannot be excluded [50].

## 5. Conclusions

In this work, we determined the chemical composition, enantiomeric distribution, AEDA profile, and enzymatic inhibition of AChE and BChE of the essential oil of *Coreopsis triloba* S.F. Blake for the first time. Twenty-nine compounds, representing 98.3–99.5% of the EO were identified. The main components were(*E*)-*β*-ocimene (35.2–35.9%), followed by *β*-phellandrene (24.6–25.0%), α-pinene (15.3–15.9%), myrcene (10.9–11.0%), sabinene (2.2–2.4%), (*Z*)-*β*-ocimene (1.5%), and germacrene D (1.2–1.3%). The enantioselective analysis indicated that *(+)-β*-pinene and (−)-germacrene D were present as pure enantiomers, while (*S*)-*α*-pinene and (*S*)-limonene presented the enantiomeric excess of 63.2% and 95.0% respectively. According to GC-O, the enantiomeric mixture of *α*-pinene was the main component responsible for the aroma of the essential oil, followed by *β*-pinene (0.6%), terpinolene (0.1%), *α-*copaene (0.1–0.3%), *β-*phellandrene, and (*E*)-4,8-dimethyl-1,3,7-nonatriene (0.1–0.2%). The essential oil showed an IC_50_ inhibition value of 42.2 µg/mL for AChE and 6.8 µg/mL for BChE.

We can conclude that *C. triloba* is a source (0.1% w/w of fresh plant material) of a new monoterpene based EO, characterized by an interesting, quite selective inhibition activity of BChE.

The secondary metabolites responsible for the herbaceous/spicy odor of the whole essential oil were also determined.

## Figures and Tables

**Table 1:** Chemical composition of *Coreopsis triloba* S.F. Blake essential oil.

Component	DB-5MS		HP-INNOWAX		DB-5MS		HP-INNOWAX	
	LRI ^a^	LRI ^b^	LRI ^a^	LRI ^b^	% ^c^	δ ^d^	% ^c^	δ ^d^
*α*-pinene	925	932 [21]	1017	1025 [22]	15.9	0.88	15.3	0.78
sabinene	965	969 [21]	1118	1122 [22]	2.2	0.02	2.4	0.03
*β*-pinene	968	974 [21]	1105	1110 [22]	0.6	0.03	0.6	0.03
myrcene	987	988 [21]	1163	1161 [22]	10.9	1.25	11.0	2.10
*α*-phellandrene	1002	1002 [21]	1161	1168 [22]	0.1	0.03	Trace	-
*p*-cymene	1020	1020 [21]	1269	1270 [22]	0.7	0.24	0.7	0.26
*β*-phellandrene	1025	1025 [21]	-	-	24.6	0.05	25.0	0.08
limonene	-	-	1196	1198 [22]	-	-	1.5	0.10
(*Z*)-*β*-ocimene	1034	1032 [21]	1236	1235 [22]	1.5	0.09	1.5	0.10
(*E*)-*β*-ocimene	1046	1044 [21]	1253	1250 [22]	35.9	1.73	35.2	2.08
*γ*-terpinene	1053	1054 [21]	1205	1245 [22]	Trace	-	0.1	0.01
terpinolene	1079	1086 [21]	1280	1278 [23]	0.1	0.01	0.1	0.01
(*E*)-4,8-dimethyl-1,3,7-nonatriene	1112	1117 [24]	1309	1309 [25]	0.1	0.01	0.2	0.01
*α*-copaene	1362	1374 [21]	1481	1489 [26]	0.1	0.02	0.3	0.05
*β*-cubebene	1376	1387 [21]	1531	1542 [22]	0.3	0.04	0.3	0.05
(*E*)-caryophyllene	1403	1417 [21]	1585	1599 [22]	0.2	0.03	0.2	0.03
*α*-humulene	1439	1452 [21]	1657	1667 [22]	0.1	0.02	0.1	0.03
*cis*-cadina-1(6),4-diene	1465	1461 [21]	1697	-	0.7	0.08	0.7	0.11
*γ*-curcumene	1469	1481 [21]	-	-	Trace	-	-	-
*ar*-curcumene	1473	1479 [21]	1770	1770 [27]	0.2	0.03	Trace	-
germacrene-D	1479	1480 [21]	1722	1727 [26]	1.3	0.21	1.2	0.22
bicyclogermacrene	1495	1500 [21]	1729	1730 [26]	Trace	-	Trace	-
*β*-bisabolene	1499	1505 [21]	-	-	0.2	0.02	-	-
*δ*-cadinene	1506	1522 [21]	1750	1756 [22]	0.8	0.25	0.6	0.48
spathulenol	1562	1577 [21]	2118	2127 [22]	0.8	0.53	0.9	0.55
cayophyllene oxide	1564	1582 [21]	1968	1986 [22]	0.5	0.44	0.5	0.40
eremoligenol	1627	1629 [21]	2179	2205 [28]	0.5	0.16	0.5	0.15
elemol	-	-	2078	2080 [27]	-	-	0.5	0.50
*γ*-eudesmol	-	-	2164	2176 [22]	-	-	0.1	0.12
**Monoterpene hydrocarbons**					**92.5**		**93.4**	
**Sesquiterpene hydrocarbons**					**4.0**		**3.6**	
**Oxygenated sesquiterpene**					**1.8**		**2.5**	
**Total amount of compounds**					**98.3**		**99.5**	

^a^ Calculated linear retention index (LRI); ^b^ reference linear retention index; ^c^ content; ^d^ standard deviation; trace% < 0.1.

**Table 2:** Enantiomeric excess of some constituents from *Coreopsis triloba* S.F. Blake.

Component	RT ^a^ (min)	LRI ^b^	Enantiomeric Distribution (%)	*e.e.* %
(*S*)-*α*-pinene	13.68	932	81.6	63.2
(*R*)-*α*-pinene	13.81	934	18.4	
(+)-*β*-pinene	15.31	959	100.0	100.0
(*R*)-limonene	21.63	1063	2.5	95.0
(*S*)-limonene	22.58	1078	97.5	
(−)-germacrene D	45.16	1474	100.0	100.0

^a^ Retention time (RT); ^b^ calculated on diethyl tertbutylsilyl-β-cyclodextrin chiral stationary phase.

**Table 3:** Components of the olfactory profile of *Coreopsis triloba* S.F. Blake essential oil.

Odor	AEDA (FD)	Compound	Calculated LRI
Woody, herbaceous	6	*α*-pinene	925
Woody	3	*β*-pinene	968
Herbaceous, green	2	*β-*phellandrene	1025
Herbaceous, sweet	3	terpinolene	1079
Spicy	2	(*E*)-4,8-dimethyl-1,3,7-nonatriene	1112
Woody, fresh	3	*α-*copaene	1362

**Table 4:** Cholinesterase inhibitory activity.

Sample	AChE, IC50, μg/mL	BChE, IC50, μg/mL
*Coreopsis triloba* S.F. Blake	42.2 ± 3.5	6.8 ± 0.6
